# Biochemical characterization of the maltokinase from *Mycobacterium bovis *BCG

**DOI:** 10.1186/1471-2091-11-21

**Published:** 2010-05-27

**Authors:** Vítor Mendes, Ana Maranha, Pedro Lamosa, Milton S da Costa, Nuno Empadinhas

**Affiliations:** 1 Center for Neuroscience and Cell Biology, University of Coimbra, 3004-517 Coimbra, Portugal; 2 Instituto de Tecnologia Química e Biológica, Universidade Nova de Lisboa, 2780-156 Oeiras, Portugal; 3 Centro de Ressonância Magnética António Xavier, Instituto de Tecnologia Química e Biológica, Universidade Nova de Lisboa, 2781-901 Oeiras, Portugal; 4 Department of Life Sciences, University of Coimbra, 3001-401 Coimbra, Portugal

## Abstract

**Background:**

Maltose-1-phosphate was detected in *Mycobacterium bovis *BCG extracts in the 1960's but a maltose-1-phosphate synthetase (maltokinase, Mak) was only much later purified from *Actinoplanes missouriensis*, allowing the identification of the *mak *gene. Recently, this metabolite was proposed to be the intermediate in a pathway linking trehalose with the synthesis of glycogen in *M. smegmatis*. Although the *M. tuberculosis *H37Rv *mak *gene (Rv0127) was considered essential for growth, no mycobacterial Mak has, to date, been characterized.

**Results:**

The sequence of the Mak from *M. bovis *BCG was identical to that from *M. tuberculosis *strains (99-100% amino acid identity). The enzyme was dependent on maltose and ATP, although GTP and UTP could be used to produce maltose-1-phosphate, which we identified by TLC and characterized by NMR. The K_*m *_for maltose was 2.52 ± 0.40 mM and 0.74 ± 0.12 mM for ATP; the *V*_max _was 21.05 ± 0.89 μmol/min.mg^-1^. Divalent cations were required for activity and Mg^2+ ^was the best activator. The enzyme was a monomer in solution, had maximal activity at 60°C, between pH 7 and 9 (at 37°C) and was unstable on ice and upon freeze/thawing. The addition of 50 mM NaCl markedly enhanced Mak stability.

**Conclusions:**

The unknown role of maltokinases in mycobacterial metabolism and the lack of biochemical data led us to express the *mak *gene from *M. bovis *BCG for biochemical characterization. This is the first mycobacterial Mak to be characterized and its properties represent essential knowledge towards deeper understanding of mycobacterial physiology. Since Mak may be a potential drug target in *M. tuberculosis*, its high-level production and purification in bioactive form provide important tools for further functional and structural studies.

## Background

The loss of human lives for tuberculosis (TB) remains unhampered as a result of the synergy with the AIDS pandemic and the emergence of drug resistant strains. Each year, there are almost 2 million TB-related deaths worldwide and nearly 9 million people become infected. Moreover, one third of the world's population is estimated to be infected with TB [1]. The available therapies target only a few bacterial functions and the growing numbers of extensively drug-resistant strains urges for the identification of novel pathways and new drug targets. While promising antimycobacterials have been recently synthesized, they are still far from being available for inclusion in TB therapies [2-4].

Maltose-1-phosphate was initially identified in *Mycobacterium bovis *BCG cell extracts [5]. An enzyme synthesizing maltose-1-phosphate from maltose and ATP was later identified in *Actinoplanes missouriensis *and named maltokinase (Mak) [6]. Apparently, it was constitutively expressed, regardless of the sugars present in the growth medium, thus unlikely to play an essential role in maltose catabolism [7]. A recombinant Mak from *Streptomyces coelicolor *was also partially characterized [8]. Maltose-1-phosphate could also be synthesized without the requirement of ATP in *E. coli*, where it was proposed to be involved in the regulation of maltose metabolism [9]. The maltokinase gene (*mak*) is present in most of the available mycobacterial genomes and it was considered essential for the growth of *M. tuberculosis *H37Rv [10]. Moreover, *mak *is always linked with the trehalose synthase gene (*treS*) and in some organisms fused into one gene. This highly conserved sequential arrangement is found in many bacterial groups and suggests a shared metabolic route.

Trehalose is a ubiquitous disaccharide found in bacteria, archaea, fungi, plants and in some invertebrates, where it plays a multitude of biological roles [11,12]. In mycobacteria and related organisms, trehalose is synthesized by three different pathways and serves a functional role as a compatible solute under osmotic or thermal stress, and a structural role in cell wall components [13-15]. *Mycobacterium smegmatis *mutants defective in the three pathways are unable to grow unless trehalose in supplemented to the growth medium [14]. However, each pathway seems to have a specific role and hierarchy in closely related actinobacterial species. While the OtsA/OtsB pathway seems to be essential for the survival of *M. tuberculosis *in infected mice, the inactivation of the TreY/TreZ did not cause obvious phenotypic effects under the examined conditions [16]. However, the inactivation of TreS promoted increased survival of the mutant in mice, when compared to the wild-type strain [16]. In *Corynebacterium glutamicum*, the TreY/TreZ pathway dominates, the OtsA/OtsB plays only a minor accessory role and the TreS pathway seems to be involved in trehalose degradation [17,18]. Single mutations targeting each of the pathways in *M. smegmatis *resulted in no apparent phenotypic defects indicating that the three pathways are redundant in this organism [14].

The TreS from *M. smegmatis *is capable of interconverting trehalose and maltose [19]. TreS was also shown to catalyze the formation of trehalose from glycogen via its amylase activity and with maltose as the intermediate [20]. Moreover, the synthesis of glycogen from trehalose has also recently been reported in *M. smegmatis *[21]. In this proposed three-step pathway, TreS and Mak sequentially convert trehalose into maltose and further into maltose-1-phosphate while a final maltosyltransferase transfers maltose from maltose-1-phosphate to glycogen. Although *M. smegmatis *has an essential trehalase to degrade trehalose directly into glucose [22], it has been suggested that it is energetically advantageous to convert trehalose to glycogen rather than to hydrolyze it to glucose [21], which cannot accumulate in cells.

Despite of the increasing numbers of genome sequences showing that *mak *genes are widely distributed throughout the bacterial world, there is still a tremendous lack of biochemical data on the corresponding enzymes as only the Mak from *A. missouriensis *and *Streptomyces coelicolor *have been biochemically characterized [7,8]. Therefore, we cloned and successfully expressed in *E. coli *the *mak *gene from *M. bovis *BCG, the organism where maltose-1-phosphate was firstly detected. We purified the recombinant bioactive enzyme and determined its biochemical and kinetic properties to further understand its role in mycobacterial physiology.

## Results

### Identification of mycobacterial maltokinase (Mak) and sequence analysis

**Figure 1:**
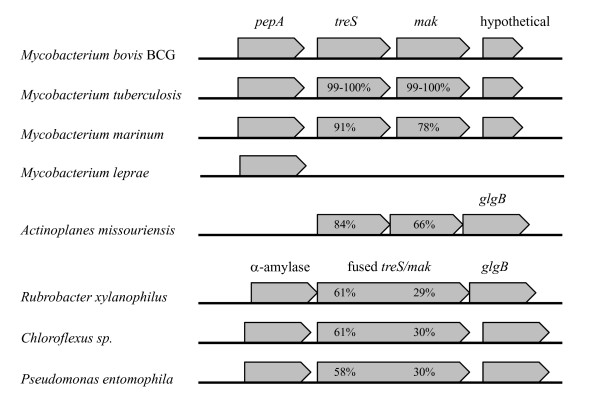
**Genetic environment of the *mak *gene in different organisms**. Organization of the region containing the *mak *gene in *Mycobacterium bovis *BCG, *M. tuberculosis*, *M. marinum*, *M. leprae*, *Actinoplanes missouriensis*, *Rubrobacter xylanophilus*, *Chloroflexus *sp. and *Pseudomonas entomophila*. Arrows represent genes and their orientation. *pepA*, serine protease; *treS*, trehalose synthase; *mak*, maltokinase; *glgB*, glycogen branching enzyme; fused *treS/mak*, probable fused trehalose synthase/maltokinase.

BLAST analysis with the *Actinoplanes missouriensis *and *Streptomyces coelicolor *maltokinase sequences within mycobacterial genome databases showed that the *mak *gene was annotated as a hypothetical protein/trehalose synthase fused maltokinase gene with homologues in most of these genomes. The amino acid identities of these proteins to the *A. missouriensis *sequence were between 62% and 67%, and 39% to 41% to the *S. coelicolor *Mak sequence. The maltokinase gene (*mak*) from *M. bovis *BCG contained 1368 bp coding for a polypeptide with 455 amino acids with a calculated molecular mass of 49.9 kDa and a calculated isoelectric point of 5.2. The Mak protein was 99 to 100% identical to the homologues from all *M. tuberculosis *and *M. bovis *strains with the genomes available (Fig. 1). Additional Mak homologues were found in *M. avium *(79% amino acid identity), *M. kansasii *(79%), *M. marinum *(79%), *M. intracellulare *(78%), *M. ulcerans *(78%), *M. smegmatis *(64%), *M. gilvum *(60%) and *M. vanbaalenii *(59%), but not in *M. leprae *nor in *M. abscessus*, which also lack *treS *genes. In other organisms like *Rubrobacter xylanophilus *or *Pseudomonas fluorescens *the *mak *gene is fused with the *treS *gene (Fig. 1). In *M. bovis*, *M. tuberculosis *and *M. marinum *the *mak *and *treS *genes have intergenic regions of about 72 to 102 bp that could accommodate a promoter sequence, implying independent regulation of *mak *expression. Our attempts to detect consensus promoter motifs in this region indicated about 69 to 75% probability of these elements within the 3' end of the *treS *gene (Fig. 1).

The Mak protein had significant sequence similarity with putative aminoglycoside phosphotransferases from several organisms of the phylum *Actinobacteria*, namely *Kribbella flavida *(NCBI accession number YP_003382767, 49% amino acid identity), *Actinosynnema mirum *(YP_003103727, 45% identity) and *Nakamurella multipartita *(YP_003202415, 45% identity).

### Expression and properties of the recombinant maltokinase

**Figure 2:**
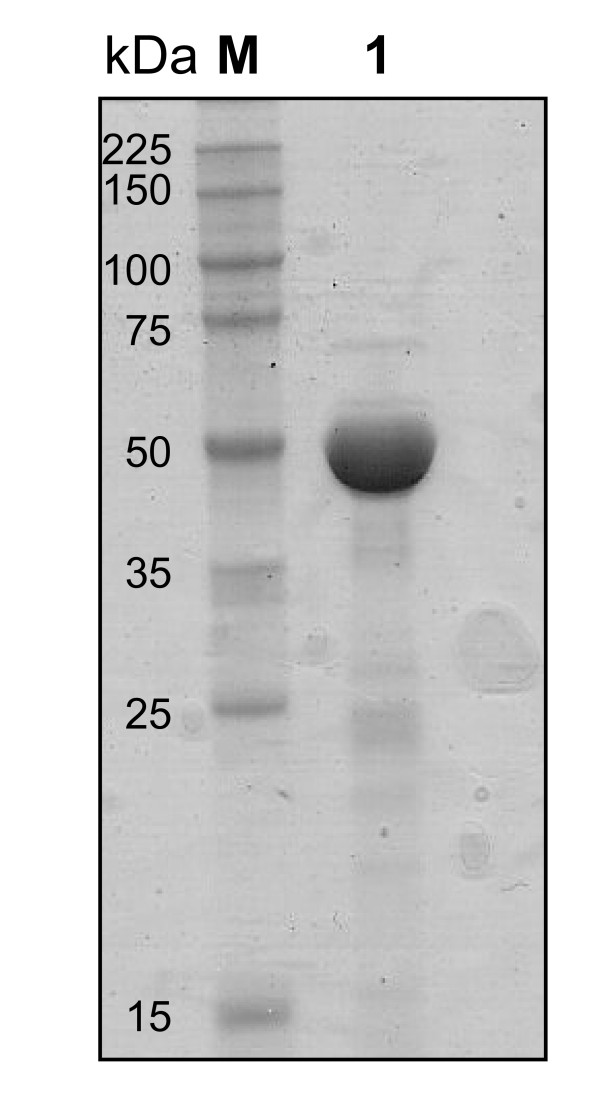
**SDS-PAGE showing the recombinant maltokinase (Mak) from *M. bovis *BCG**. Lane 1 - Purified recombinant Mak. M - Protein molecular weight markers.

Expression of the *M. bovis *BCG *mak *gene in *E. coli *resulted in the high level production of recombinant His-tagged protein (Fig 2). The Mak identity was confirmed by peptide mass fingerprinting. Gel filtration experiments indicated that the recombinant His-tagged Mak behaved as a monomeric protein in solution, with a molecular mass of about 50.7 ± 4.2 kDa.

**Figure 3:**
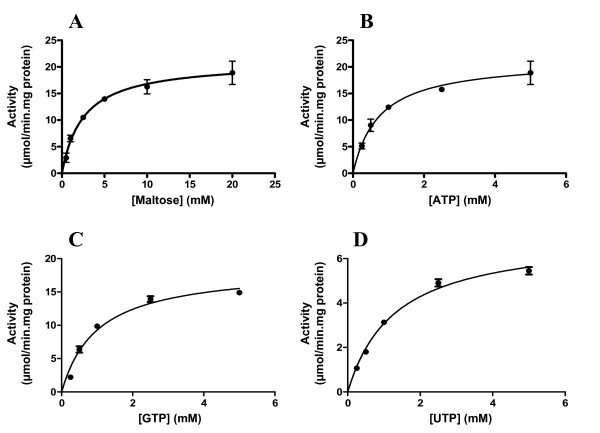
**Kinetic studies of Mak activity**. Dependence of Mak activity on the concentrations of maltose (A), ATP (B), GTP (C) and UTP (D).

The recombinant Mak used ATP, GTP and UTP as phosphate donors with comparable but decreasing efficiency (Table 1). Residual activity was also detected with ADP and GDP (< 7%). Among the sugar substrates tested, maltose was, by far, the preferred acceptor. Residual activity was also detected with maltotriose, maltoetraose, maltopentaose and maltoheptaose (< 2%), which may be due to maltose contamination. Due to the sequence similarities of Mak with putative aminoglycoside phosphotransferases, the aminoglycoside antibiotics gentamicin, hygromycin B, kanamycin and streptomycin were also tested as possible acceptors of phosphate but no activity was detected. Kinetic experiments showed that the recombinant Mak exhibited Michaelis-Menten kinetics at 37°C with ATP, GTP and UTP up to 5 mM (Table 1 and Fig. 3). Higher concentrations of these phosphate donors were progressively inhibitory, in the reaction mixture containing 20 mM maltose and 10 mM MgCl_2_. While maximum Mak activity was observed with 5 mM ATP, the activity dropped to 75% with 10 mM ATP and to about 50% with 20 mM ATP.

**Table 1:** Kinetic parameters of the recombinant maltokinase from *Mycobacterium bovis *BCG

Substrate	Maltose	ATP	GTP	UTP
K_*m *_(mM)	2.52 ± 0.40	0.74 ± 0.12	1.01 ± 0.17	1.30 ± 0.13

*V*_*max*_(μmol/min.mg^-1^)	21.05 ± 0.89	21.42 ± 1.03	18.65 ± 1.10	7.07 ± 0.28

**Figure 4:**
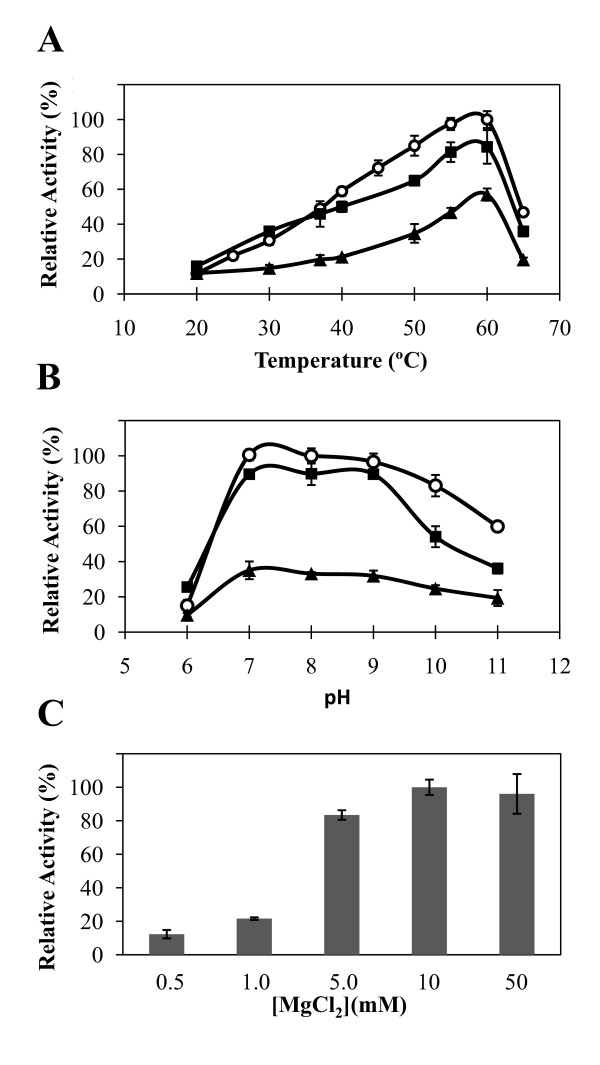
**Effects of temperature, pH and Mg^2+ ^concentration on Mak activity**. Temperature profile (A) and pH dependence (B) using ATP (white circle), GTP (black circle) and UTP (black triangle) as phosphate donors. (C) Effect of Mg^2+ ^concentration.

The enzyme was active between 20 and 65°C, with maximal activity at about 60°C (Fig. 4A). At 37°C, the enzyme was active between pH 6 and 11, optimally between pH 7 and 9 with maltose and ATP, GTP or UTP (Fig. 4B). The recombinant Mak was strictly dependent on divalent cations with Mg^2+ ^(10 mM) having the most pronounced stimulatory effect (Fig. 4C). Other divalent cations like Co^2+ ^and Mn^2+ ^also activated Mak, but to lower extent (~41% and ~14% of maximal activity, respectively). The enzyme retained only about 40% of activity after 7 days storage at 4°C in 50 mM BTP (pH 7.5). Most of the activity was also lost after freeze/thawing the enzyme at -20°C in the same buffer. Glycerol strongly inhibited Mak activity, even at very low concentrations (1%). The addition of 10 mM maltose (final concentration) only slightly improved the stability at 4°C to about 50% of maximal activity. However, the addition of 50 mM NaCl dramatically improved the stability of the enzyme, as the residual activity after 1 week at 4°C was still above 90% of maximal activity.

### Identification of maltose-1-phosphate by NMR

**Figure 5:**
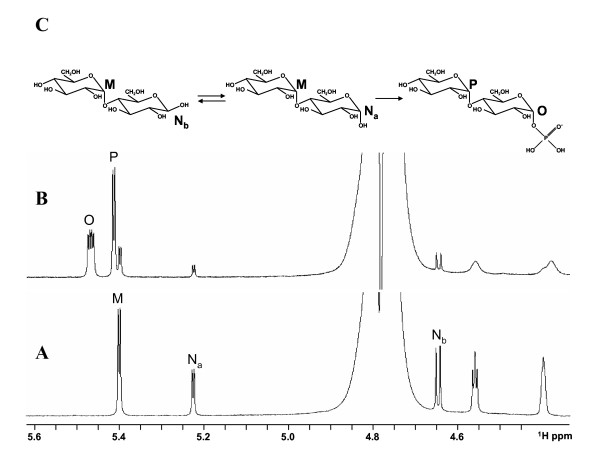
**^1^H-NMR spectra of the Mak reaction**. Mixture contained 5 mM each ATP and maltose, 5 mM MgCl_2 _in 10 mM BTP at pH 8, prior (A) and after (B) the addition of 15 μg of Mak and incubation for 10 min at 37°C. The top panel (C) represents the structures of maltose and maltose-1-phosphate and is labeled according to their assigned resonances.

Maltose is composed of two glucose residues linked via an alpha-1,4-glycosidic bond. This means that one of the residues is blocked in the alpha configuration, while the other has a free aldehyde group at position one and therefore can adopt either the alpha or the beta configuration (Fig. 5C). This explains the presence of the three signals in the ^1^H-NMR spectrum of maltose (Fig. 5A), in the region where protons 1 of hexoses resonate; signal M corresponding to proton 1 of the blocked residue and signals N_a _and N_b _corresponding to proton 1 of unblocked residue in the alpha and beta configurations, respectively. After incubating the reaction mixture with the enzyme (Fig 5B), signals M, N_a_, and N_b _almost disappear and only two new signals arise (signals O and P). These signals present H-H coupling constants ^3^J_1,2 _around 3 Hz, meaning that the respective residues are in the alpha configuration. Additionally, resonance O presents a second 7 Hz splitting of the signal, consistent with a H-P coupling constant. This phosphorylation was confirmed by a ^1^H-^31^P HSQC spectrum that presents a correlation between signal O and a phosphorus resonance at 1.64 ppm (the region where phosphomonoesters resonate). This means that the reaction product has two hexose residues blocked in the alpha configuration and that one of them is phosphorylated at position 1, thus we conclude to be in the presence of alpha-D-maltose-1-phosphate. This result was further confirmed by the acquisition of a ^1^H-^13^C HSQC spectrum and comparison of the ^1^H and ^13^C resonances with the literature [6].

## Discussion

Maltose-1-phosphate was identified for the first time in *Mycobacterium bovis *BCG [5] but a maltose-1-phosphate synthesizing enzyme (maltokinase, Mak) was only identified much later in *Actinoplanes missouriensis *[6]. Although the corresponding gene (*mak*) can now be identified in many bacterial genomes, including those of most mycobacteria, only the Mak from *A. missouriensis *and *S. coelicolor *have, so far, been characterized [7,8]. Interestingly, the *M. tuberculosis *H37Rv *mak *gene (Rv0127) was considered essential for the pathogen's growth [10]. This information and the absence of biochemical data on mycobacterial maltokinases prompted us to study the enzyme from the slow-growing species *M. bovis *BCG.

Our results show that, in contrast to the *A. missouriensis *and *S. coelicolor *enzymes, which use ATP as the sole phosphate donor (GTP and UTP are used at less than 3% by the *A. missouriensis *Mak and not used by the *S. coelicolor *enzyme), the *M. bovis *BCG maltokinase was able to use ATP, GTP and UTP with comparable efficiency. This substrate flexibility may reflect an absolute requirement for maltose-1-phosphate, corroborating the proposed essentiality of the *mak *gene in *M. tuberculosis *[10]. However, lower flexibility of the Mak for NTP substrates may occur *in vivo*. Similar behavior has been detected between native and recombinant forms of enzymes involved in trehalose metabolism [23,24]. Interestingly, some unprecedented residual activity (< 7%) could also be detected with ADP and GDP as phosphate donors. Like the enzymes from *A. missouriensis *and *S. coelicolor*, only maltose served as the acceptor substrate. Although the K_*m *_values for maltose were similar for the *A. missouriensis *and *M. bovis *BCG Mak (no kinetic data is available for the *S. coelicolor *Mak), the latter had a K_*m *_value for ATP (0.74 mM) slightly higher than that measured for the *A. missouriensis *Mak (0.54 mM).

A major obstacle for the characterization of the *M. bovis *BCG maltokinase was its very low stability. Our attempts to stabilize the enzyme with glycerol failed as it severely inhibited the activity. Unlike the native Mak from *A. missouriensis*, which could be stabilized by maltose, this disaccharide only moderately stabilized the mycobacterial Mak while trehalose was ineffective. Only the addition of 50 mM NaCl to the preparation stabilized the enzyme for characterization. The mycobacterial Mak was, like the *A. missouriensis *and the *S. coelicolor *enzymes, a monomer in solution. While no data is available for the cation dependence of the *S. coelicolor *Mak, both the *A. missouriensis *and the *M. bovis *BCG enzymes were dependent on Mg^2+ ^ions for maximal activity. The cations Co^2+ ^or Mn^2+ ^could partially replace Mg^2+ ^while Zn^2+ ^was inhibitory. Divalent cations seem to play an essential role in the activity of kinases, namely in the stabilization of the negatively charged phosphate donor groups [7]. The temperature profile for activity of the recombinant Mak from *M. bovis *BCG was similar to that of the native Mak from *A. missouriensis*, with maximum near 60°C. The *S. coelicolor *recombinant Mak had maximal activity at about 45°C. All three enzymes had optimal activity between pH 7 and 9 [7,8], which is also the optimum pH range for the activity of other kinases [7]. The Mak from *M. bovis *BCG had no detectable aminoglycoside phosphotransferase activity, which is in agreement with the data obtained with the *S. coelicolor *Mak [8]. It is likely that many of the putative aminoglycoside phosphotransferases with significant amino acid identity with maltokinases (>40%) are incorrectly annotated and may instead have maltokinase activity.

**Figure 6:**
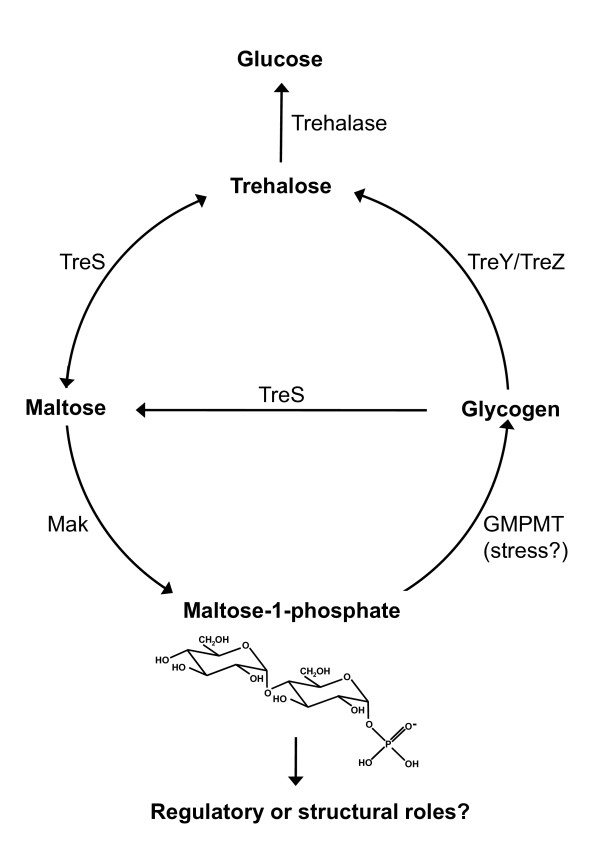
**Metabolic circuit between trehalose, maltose and glycogen and proposal of alternative roles for maltose-1-phosphate**. Data adapted from references [13,20,21]. Mak, maltokinase, TreS, trehalose synthase, TreY/TreZ, maltooligosyltrehalose synthase/trehalohydrolase, GMPMT, α-1,4-glucan: maltose-1-P maltosyltransferase.

The frequent association of the *mak *gene with the trehalose synthase (*treS*) gene, either as a bicystronic unit or as a fused gene, strongly suggests a synergistic action and a common biochemical pathway. While such a pathway has been previously proposed [8], very recent research [21] corroborate this hypothesis by demonstrating that *M. smegmatis *converts excess trehalose into glycogen through the sequential action of TreS, Mak and of a maltosyltransferase (Fig. 6). The latter enzyme was shown to use maltose-1-phosphate as the substrate for the transfer of maltose to glycogen [21]. The genetic organization in *A. missouriensis *lend additional support to this hypothesis since a glycogen-branching enzyme gene (*glgB*) is located immediately downstream the contiguous *treS *and *mak *genes. In *Rubrobacter xylanophilus*, a bacterium that constitutively accumulates high levels of trehalose, a *glgB *gene is also located immediately downstream a fused *treS*-*mak *bifunctional gene and a similar function may be anticipated. Interestingly, the *M. smegmatis *TreS was also found to possess amylase activity and capable to catalyze the synthesis of trehalose from glycogen, with maltose as intermediate [20]. Curiously, *Mycobacterium leprae*, in which glycogen has not been reported, lacks both *treS *and *mak *homologues [25]. However, the reason for the missing glycogen may be explained by the absence of other genes involved in glycogen synthesis like *glgB *or *glgP*, which are pseudogenes in *M. leprae*. On the other hand, both *M. gilvum *and *M. vanbaalenii *possess a *mak *but lack a *treS *gene. Unfortunately no data on trehalose or glycogen synthesis is available for these organisms. Nevertheless, among the mycobacterial enzymes involved in this metabolic circuit (trehalose → maltose → maltose-1-phosphate → glycogen → maltose → trehalose) (Fig. 6), all enzymes but the maltokinase have been biochemically studied [13,19,21].

Mycobacteria possess three different pathways for the synthesis of trehalose [13,16]. This disaccharide is constitutively present in the cytoplasm of mycobacterial cells, an essential structural component of cell walls and its levels increase during osmotic and thermal stress [13-15]. Although the TreS has much higher affinity for maltose than for trehalose [19], in most cases it seems to be involved in trehalose degradation [16] and has only been implicated in trehalose biosynthesis in *M. smegmatis *[14]. *M. smegmatis *possesses an essential trehalase that converts trehalose into glucose [22] (Fig. 6). Elbein and coworkers argue that it is energetically favorable to convert trehalose to glycogen by the recently discovered TreS-Mak-maltosyltransferase pathway (Fig. 6) [21]. However, normal ATP intracellular concentrations within the 1 to 10 mM range [26] are inhibitory of the maltosyltransferase (GMPMT) activity [21] but not of the Mak activity. These authors also propose that the role of this pathway is to convert excess trehalose into glycogen during stress conditions. Therefore, under non-stressing conditions, in which the ATP levels rise, maltose-1-phosphate cannot be the substrate for glycogen, which may be synthesized via ADP-glucose [21]. Since mycobacteria contain other alpha-1,4-glucans and polysaccharides [25], it is tempting to speculate that maltose-1-phosphate may also be used as donor of maltose units for those macromolecules, under specific conditions (Fig. 6). Moreover, the constitutive expression of Mak in *A. missouriensis *[7] and the suggested essentiality of the *M. tuberculosis mak *gene (Rv0127) indicate alternative roles for maltose-1-phosphate in mycobacterial physiology. This is also supported by the fact that only *mak*, but not the *treS *gene (Rv0126), was considered essential for growth. Moreover, the presence of a putative promoter within the terminal part of the *treS *gene indicating independent regulation of *mak *expression, also suggests an alternative role for the Mak and maltose-1-phosphate in mycobacteria. Since trehalose-6-phosphate is an important signaling molecule in yeast and plant metabolism [12,27], maltose-1-phosphate could, hypothetically, play a similar role in mycobacteria. In fact, the involvement of maltose-1-phosphate in the regulation of sugar metabolism in *E. coli *has been proposed earlier but not confirmed experimentally [9].

Several questions remain unanswered and the role(s) of maltose-1-phosphate in mycobacterial metabolism only now begins to be unraveled. While further studies are required to answer these questions, the biochemical properties of the maltokinase from *M. bovis *BCG, the first of this bacterial group to be characterized, provide an important step towards the clarification of its role in mycobacterial metabolism.

## Conclusions

Although the maltokinase gene was considered essential for *M. tuberculosis *growth [10], implying that the corresponding enzyme might be a suitable target for the design/development of anti-mycobacterial drugs, no mycobacterial maltokinase had been characterized to date. The fast and straightforward method for high-level production and purification of bioactive maltokinase from *M. bovis *BCG, a major bottleneck in mycobacterial research, and the determination of its biochemical properties, some of which are unique among these type of enzymes, contribute to the knowledge of these enzyme's abilities and provide insights about its unexplored role in mycobacterial physiology.

## Methods

### Identification, sequence analysis, cloning and functional overexpression of the maltokinase gene (mak) from *Mycobacterium bovis *BCG, strains and growth conditions

To identify the *mak *gene in mycobacterial genomes we used the amino acid sequences of the *Actinoplanes missouriensis *and *Streptomyces coelicolor *maltokinases (GenPept accession number AAQ01690 and CAA04602, respectively) in BLAST searches at the National Center for Biotechnology Information (NCBI, http://blast.ncbi.nlm.nih.gov/Blast.cgi) database. Promoter identification was carried out using the prokaryotic promoter prediction software from Berkeley University, available at http://www.fruitfly.org. The *mak *gene was amplified from the chromosomal DNA from *M. bovis *BCG (DSM 43990) obtained from the Deutsche Sammlung von Mikroorganismen und Zellkulturen GmbH (DSMZ, Braunschweig, Germany), with a pair of primers based on the gene sequence retrieved from the Institut Pasteur database http://genolist.pasteur.fr. An NdeI restriction site (highlighted in bold) was added to the forward primer MtuNde (5'-CTTA**CATATG**ACTCGGTCGGACACGC-3'). A HindIII restriction site (highlighted in bold) was added to the reverse primer MtuHind (5'-ATT**AAGCTT**GCTAGCGGTCAGGCGGG-3'). The stop codon was removed from the reverse primer to allow the translation of a C-terminal 6 × His-tag encoded by the expression vector pET30a (Novagen). PCR was carried out with the AccuPrime GC-Rich DNA Polymerase (Invitrogen). DNA (200 ng) was denatured by 95°C for 5 min followed by 30 cycles of 1 min denaturation step at 95°C, 1 min annealing step at 62°C, and 1.5 min extension step at 72°C. The PCR product was purified from agarose gel (NZYTech, Portugal), digested and cloned into pET30a. The construct was sequenced to confirm the identity of the insert (AGOWA, Berlin, Germany), and transformed into *E. coli *BL-21, which was used as host for expression of the *mak *gene. Recombinant *E. coli *was grown in a 5L fermentor, with continuous aeration and stirred at 180 rpm at 37°C, pH 7.0 in LB medium with kanamycin (30 μg/ml), to mid-exponential phase of growth (OD610 = 0.8). IPTG was added at a final concentration of 0.5 mM to induce gene expression, and temperature was reduced to 20°C. The cells were harvested 18 h later by centrifugation (9000 × *g*, 10 min, 4°C).

### Preparation of cell-free extracts

*Escherichia coli *cells carrying the recombinant maltokinase (Mak) from *M. bovis *BCG were suspended in 25 mM Bis-tris propane buffer (BTP) at pH 7.5 with 50 mM NaCl for enzyme assays, or in 20 mM sodium phosphate buffer at pH 7.4 with 0.5 M NaCl and 20 mM imidazole, for protein purification. A protease inhibitor cocktail (Roche), 10 μg/ml DNAse I and 5 mM MgCl_2 _were added to the suspension. Cells were disrupted twice in a French-press cell followed by centrifugation (15000 × *g*, 4°C, 30 min).

### Enzyme assays

The activity of the recombinant Mak from *M. bovis *BCG in *E. coli *extracts and during purification was detected after 15 min at 37°C in reaction mixtures (50 μl) containing 25 μl of cell-free extract, 3.0 mM (each) of ATP and maltose, and 10 mM MgCl_2 _in 50 mM BTP, pH 8.0. The synthesis of maltose-1-phosphate was monitored by thin-layer chromatography (TLC) with solvent systems composed by acetic acid/ethyl acetate/water/ammonia 25% (6:6:2:1, v/v) and butanol/ethanol/water (5:3:2, v/v). Trehalose-6-phosphate, ATP, GTP, UTP, ADP, GDP, AMP and maltose standards were used for comparative purposes. The maltose-1-phosphate formed was identified by NMR as described below. *E. coli *cell-free extracts carrying an empty vector were used as negative controls. Protein concentration was determined by the Bradford method [28].

### Purification of recombinant Mak from *M. bovis *BCG

The His-tagged recombinant Mak from *M. bovis *BCG was purified in a prepacked Ni-Sepharose high-performance column (His-Prep FF 16/10) equilibrated with 20 mM sodium phosphate, pH 7.4, 0.5 M NaCl, and 20 mM imidazole. Elution was carried out with 500 mM imidazole and the purity of the fractions was determined by SDS-PAGE. The purest active fractions were pooled, diluted ten times with 25 mM BTP at pH 7.4 and loaded into a Q-Sepharose fast-flow column (Hi-Load FF 16/10), equilibrated with 25 mM BTP at pH 7.4 with 50 mM NaCl, and eluted by a linear gradient of NaCl (50 to 500 mM). The purity of the fractions was determined by SDS-PAGE and the purest active fractions were pooled, concentrated by ultracentrifugation in 30 kDa cutoff centricons (Amicon), equilibrated with 25 mM BTP at pH 7.4 with 200 mM NaCl, and loaded into a Superdex 200 fast-flow column equilibrated with the same buffer. After SDS-PAGE analysis the active pure fractions were concentrated and equilibrated with 50 mM BTP at pH 7.4 with 50 mM NaCl. Protein content of the samples was determined by the Bradford assay [28]. The identity of the purified maltokinase from *M. bovis *BCG was confirmed by Peptide Mass Fingerprinting (IPATIMUP Proteomics Unit, Porto, Portugal).

### Characterization of the recombinant Mak from *M. bovis *BCG

The substrate specificity of the recombinant Mak from *M. bovis *BCG was determined using glucose, galactose, mannose, maltose, isomaltose, trehalose, maltotriose, maltotetraose, maltopentaose and maltoheptaose as possible acceptors and with ADP, CDP, GDP, TDP, UDP, ATP, CTP, GTP, TTP and UTP as possible phosphate donors (all from Sigma-Aldrich). Since the Mak protein had high sequence identity (>40%) with putative aminoglycoside phosphotransferases, this activity was also tested using the aminoglycoside antibiotics gentamicin, kanamycin, streptomycin and hygromycin B as possible phosphate acceptors (all from Sigma-Aldrich). The reaction mixtures (50 μl) containing (0.5 μg) pure recombinant Mak, 3 mM of each substrate, and 10 mM MgCl_2 _in 50 mM BTP at pH 8, were incubated at 37°C for 10 min. The products were visualized by TLC as described above.

Temperature and pH profile, effect of cations and thermal stability of Mak were determined by the addition of 0.5 μg of Mak to 50 μl reaction mixtures containing the appropriate buffer, 3 mM maltose and 3 mM NTP and stopped at different times by cooling on ethanol-ice. The Mak was inactivated by the addition of 5 μl of 1N HCl and neutralized by 5 μl of 1N NaOH. Controls were performed to account for possible NTP degradation following acid treatment. The amount of NDP released was determined at 340 nm after incubation of the sample with 3 U of pyruvate kinase and lactate dehydrogenase, 0.3 mM NADH and 2.5 mM phosphoenolpyruvate (all from Sigma-Aldrich) in 1 ml mixture (total volume) for 10 min at 30°C [29]. The temperature profile was determined between 20 and 65°C in 50 mM BTP at pH 7.0, with 10 mM MgCl_2_. The effect of pH was determined at 37°C in 50 mM BTP (pH 6.0 to 9.0) and in 50 mM CAPS (pH 9.0 to 11.0), with 10 mM MgCl_2_. The effect of cations was examined by incubating reaction mixture containing the appropriate substrates, with the chloride salts of Mg^2+^, Mn^2+^, Co^2+^, Zn^2+ ^(0.5 to 50 mM) or without cations, at 37°C.

The kinetic parameters for the recombinant Mak were determined by measuring the amount of NDP released, as described above. The *Km *values for the substrates ATP, GTP, UTP and maltose were determined at 37°C, from Lineweaver-Burk plots. All experiments were performed in triplicate.

The molecular mass of the recombinant Mak from *M. bovis *BCG was estimated by gel filtration on a Superdex 200 column and the molecular mass standards were aprotinin (6.5 kDa), ribonuclease (13.7 kDa), carbonic anhydrase (29 kDa), ovalbumine (43 kDa), conalbumine (75 kDa), aldolase (158 kDa). Blue Dextran 2000 was used to determine the void volume (Amersham).

### Enzyme stabilization assays

Pure recombinant Mak was stored in several aliquots containing 50 mM BTP at pH 8.0, and one of the following substances (final concentration) was added: 20% glycerol, 50% glycerol, 10 mM maltose, 10 mM trehalose or 50 mM NaCl. The initial Mak activity was measured at 37°C in 50 mM BTP at pH 8.0, with 10 mM MgCl_2 _and 3 mM (each) of ATP and maltose. Aliquots were stored at 4 and -20°C for seven days. Control aliquots were kept at 4° and -20°C only in 50 mM BTP at pH 8.0. After seven days storage the residual activity was measured for each of the conditions described above.

### NMR spectroscopy

For the NMR experiments, 10% (v/v) of D_2_O was added to the reaction mixture containing 5 mM (each) of ATP and maltose, 5 mM MgCl_2 _in 10 mM BTP at pH 8.0. After spectral acquisition, 15 μg of enzyme were added to the NMR tube and incubated for 10 min at 37°C. All spectra were acquired on a Bruker AVANCE III 800 spectrometer (Bruker, Rheinstetten, Germany) working at a proton operating frequency of 800.33 MHz, equipped with a four channel 5 mm inverse detection probe head with pulse-field gradients along the Z axis.

Spectra were run at 25°C using standard Bruker pulse programs. ^1^H and ^13^C chemical shifts are referenced to 3-(trimethylsilyl)propane sulfonic acid, and ^31^P resonances to external 85% phosphoric acid. In the ^1^H-^13^C and the ^1^H-^31^P heteronuclear two-dimensional single quantum coherence (HSQC) spectra, delays of 3.44 and 71.4 ms were used for evolution of the J_H, C _and J_H, P _couplings, respectively; proton decoupling was, in both cases, achieved using the GARP4 sequence [30].

## Abbreviations

Mak: maltokinase; TreS: trehalose synthase; GMPMT: α-1,4-glucan:maltose-1-P maltosyltransferase; TreY/TreZ: maltooligosyltrehalose synthase/trehalohydrolase; OtsA/OtsB: trehalose-phosphate synthase/phosphatase; *glgB*: gene for glycogen branching enzyme; *glgP*: glycogen phosphorylase gene; TLC: thin layer chromatography; NMR: nuclear magnetic resonance; SDS-PAGE: sodium dodecyl sulfate polyacrylamide gel electrophoresis; BTP: Bis-Tris propane buffer.

## Authors' contributions

VM and NE designed the concept and experiments of this study. VM cloned, expressed and purified the maltokinase. VM and AM performed the experiments for the determination of the biochemical and kinetic data. PL designed the NMR experiments and acquired the data. VM, PL and NE were involved in the analysis and interpretation of the data. VM, NE and MSC drafted the manuscript, whereas the other authors helped to draft the manuscript. All authors have approved the final manuscript.
